# Prescription Department

**Published:** 1894-01

**Authors:** 


FOR SUB-ACUTE RHEUMATISM.

℞. Potassi iodidi, ʒ iv.
Potassi bitartrat, ℥ j.
Liq. Potassi citratis, ℥ vj. M.
Sig. Shake well and take three teaspoonfuls three times a day in considerable water or in lemonade.

FOR ACUTE BRONCHITIS.

℞. Tinct. sanguinariæ.

Tinct. lobeliæ, aa ʒ j.

Vini ipecac, ʒ ij.

Syrup tolutani., ℥ iv. M.

Sig. ʒ j. every three hours.

INFANTILE CONVULSIONS.

First give a purgative dose of calomel, and follow in a few hours by
℞. Chloral Hydrat, gr. iv.
Potassi Bromidi, gr. viij.
Aquæ,
Syrupi, aa, ʒ i. M.
Sig. Dose for a child 2 years old.—*Prof. A. Jacobi.*

LOCAL ANÆSTHESIA OF THE BLADDER.

To produce local anæsthesia of the bladder inject the following:
℞. Aq. fontan. dist., f ℥ jiss.
Cocain. murat., gr. v-ix.
Sig. Sufficient for one injection.—*Il Raccoglitore Medico*, 11, 1890.

DIARRHŒA IN CHILDREN.

℞. Bismuthi subnitrat, ʒ ij.
Tinct. opii, deod., gtts. x.
Chalk mixture, q. s ℥ ij.
M. Sig. Teaspoonful every three or four hours for a child from one to five years old.

FOR HEADACHE.

℞. Papine, ℥ ss.

Caffeine cit., gr. xlviij.

Spts. amm. arom., ʒ iij.

Elix. guarana, ʒ iij.

Aq. rosæ, ʒ iij.

M. Sig. Dessertspoonful in water every hour until relieved.—*Medical Brief.*

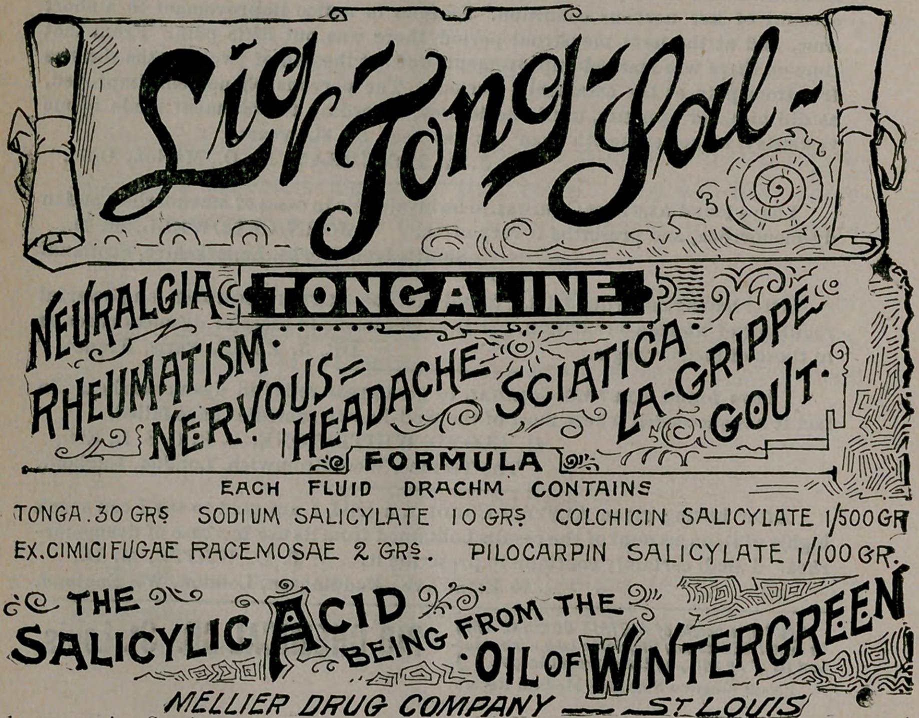

lease mention Southern Medical Record.

TREATMENT OF COLDS.

℞. Antikamnia (Genuine).
Salol.
Sulph. Quinia.
Terpin Hydrate, aa grs. xxiv.
M. ft. Capsules xii. One every four hours.—*Virginia Med. Monthly.*

AS A GENERAL TONIC IN ENDOMETRITIS:

℞. Magnesia sulphatis, ℥ii.
Ferri sulphatis, grs. xvi.
Acidi sulphurici diluti, ʒi.
Aquæ, O i.
Sig. Tablespoonful in water before meals.—*Engelmann.*

LA GRIPPE.

℞. Benzoate sodium, ½ oz.
Glycerine....... 1 oz.
Lip. Tong. Sal . 3 ozs.
Aqua Mentha Pip 2 ozs.
M. Sig. Tablespoonful every two to four hours.

ACUTE PLEURISY.

℞. Tr. Aconit. Rad., m xx.
Morphiæ Sulph., gr. i.
Ext. Ipecac. Fl., m xv.
Spts. Ether. Nitrosi, ℥i.
Aquæ, ℥i. M.
Sig. One teaspoonful every hour.—*Canada Lancet.*

ACUTE CATARRH, NASAL, PHARYNGEAL AND BRONCHIAL.

℞. Antimon. et Pot. Tart.

Morphiæ Acet., aa gr. ¼.

Aquæ, ℥ij. M.

ʒi every hour or two.—*Prof. Bartholow.*

FOR ACUTE BRONCHITIS.

℞. Terebene, f. ʒ ij.

Mucilag. acaciæ, f. ℥ij.

Morphinæ sulph., gr. ½.

Syrup tolu, f. ℥i.

M. Sig. A tenspoonful every third hour.—*Prof. Da Costa.*

